# LGR6 Is a High Affinity Receptor of R-Spondins and Potentially Functions as a Tumor Suppressor

**DOI:** 10.1371/journal.pone.0037137

**Published:** 2012-05-17

**Authors:** Xing Gong, Kendra S. Carmon, Qiushi Lin, Anthony Thomas, Jing Yi, Qingyun Liu

**Affiliations:** The Brown Foundation Institute of Molecular Medicine and Texas Therapeutics Institute, University of Texas Health Science Center at Houston, Houston, Texas, United States of America; Institute of Neurology (Edinger-Institute), Germany

## Abstract

**Background:**

LGR6 (leucine-rich repeat containing, G protein-coupled receptor 6) is a member of the rhodopsin-like seven transmembrane domain receptor superfamily with the highest homology to LGR4 and LGR5. LGR6 was found as one of the novel genes mutated in colon cancer through total exon sequencing and its promoter region is hypermethylated in 20–50% of colon cancer cases. In the skin, LGR6 marks a population of stem cells that can give rise to all cell lineages. Recently, we and others demonstrated that LGR4 and LGR5 function as receptors of R-spondins to potentiate Wnt/β-catenin signaling. However, the binding affinity and functional response of LGR6 to R-spondins, and the activity of colon cancer mutants of LGR6 have not been determined.

**Principal Findings:**

We found that LGR6 also binds and responds to R-spondins 1–3 with high affinity to enhance Wnt/β-catenin signaling through increased LRP6 phosphorylation. Similar to LGR4 and LGR5, LGR6 is not coupled to heterotrimeric G proteins or to β-arrestin following R-spondin stimulation. Functional and expression analysis of three somatic mutations identified in colon cancer samples indicates that one mutant fails to bind and respond to R-spondin (loss-of-function), but the other two have no significant effect on receptor function. Overexpression of wild-type LGR6 in HeLa cells leads to increased cell migration following co-treatment with R-spondin1 and Wnt3a when compared to vector control cells or cells overexpressing the loss-of-function mutant.

**Conclusions:**

LGR6 is a high affinity receptor for R-spondins 1–3 and potentially functions as a tumor suppressor despite its positive effect on Wnt/β-catenin signaling.

## Introduction

LGR6 (leucine rich-repeat containing, G protein-coupled receptor 6) is a member of the glycoprotein hormone receptor subfamily of rhodopsin-like, seven transmembrane domain (7TM) receptors [1]. It is most homologous to two other receptors, LGR4 and LGR5 with 50% identity between each other at the amino acid level [1]. The trio of receptors (LGR4–6) is unique in having a large N-terminal extracellular domain (ECD) containing 17 leucine-rich repeats which are flanked by cysteine-rich sequences at both the N- and C-termini. Another common feature of the three receptors is their expression in distinct types of adult stem cells [2]. LGR6 was shown to mark a group of stem cells in the skin that can give rise to all cell lineages of the skin, including those of the hair follicle, sebaceous gland, and interfollicular dermis [3]. LGR5 marks a distinct population of stem cells in the skin, which, however, only provide progenitor cells of hair follicles [4]. In the gastrointestinal tract, LGR5 marks the rapidly cycling stem cells in the crypts that can give rise to all cell types of the gut epithelium [5]. LGR4, though not a marker of adult stem cells, is generally expressed at high levels in proliferating cells of many tissues, including adult stem cells and early progenitors cells [2], [6], [7]. Importantly, LGR4 is essential for the survival and proliferation of the crypt stem cells [7], [8]. These observations suggest that LGR4–6 have unique ligands and signaling mechanisms as they are the only receptors, among hundreds of members of the rhodopsin family, found to be specifically expressed in adult stem cells and/or essential for their survival.

Recently, we and others demonstrated that LGR4 and LGR5 function as receptors of the R-spondin family of stem cell factors to potentiate Wnt/β-catenin signaling [8]–[10]. R-spondins (RSPOs) are a group of four secreted proteins (RSPO1–4) that share an overall identity of 40–60% at the amino acid sequence level and are comprised of similar domains [11]. They were originally identified as Wnt agonists based on their robust, positive effect on Wnt/β-catenin signaling [12], [13]. Stimulation of LGR4 or LGR5 with any of the four RSPOs greatly potentiates β-catenin-dependent transcription induced by Wnt3a, with RSPO2 and RSPO3 showing the highest potency and affinity [9]. Though LGR4 and LGR5 contain a 7TM domain with significant homology to those of the rhodopsin family of GPCRs, and are predicted to be G protein-coupled receptors, stimulation of neither receptor with RSPOs lead to changes in intracellular levels of cAMP or Ca^2+^, or translocation of β-arrestin [9]. Wnt/β-catenin signaling, also referred to as canonical Wnt signaling, is initiated through phosphorylation of the Wnt coreceptors LRP5/6 at multiple sites following Wnt ligand stimulation [14], [15]. One of the key phosphorylation sites is Ser-1490 of LRP6, which is greatly enhanced by co-treatment with RSPO [9], [16]. Therefore, activation of LGR4/5 by RSPOs most likely leads to increased activity of one or multiple kinases that phosphorylate LRP6 through a yet unknown mechanism.

LGR6 was shown to be able to rescue the effect of R-spondin on Wnt/β-catenin signaling in HEK293T cells when endogenously expressed LGR4 was knocked down [8], suggesting that LGR6 functions similarly as LGR4 and LGR5. However, activation of LGR6 by the different RSPOs has not yet been characterized. More importantly, LGR6 was found to be one of the commonly mutated genes in a group of colon cancer samples that were sequenced by whole-exon sequencing [17]. Out of 37 colon cancer samples randomly selected for sequencing, three mutations (299–300insGRS, G725C, and P928H) in LGR6 were found, with the mutation P928H being homozygous [17]. An independent, transcriptome-wide approach also found that the promoter region of LGR6 is hypermethylated in ∼20% of colon cancer cases [18]. A follow-up analysis showed that LGR6 is hypermethylated in ∼50% of colon cancer [19], [20], suggesting that LGR6 functions as a tumor suppressor. We mined the COSMIC (Catalogue Of Somatic Mutations In Cancer [21]) database for additional mutations and found that LGR6 is also somatically mutated in cancers of the ovary and pancreas [21]. The Wnt/β-catenin signaling pathway is well known to have critical roles in the initiation and growth of many types of cancer, especially in colon cancer, as nearly 90% of colon cancers have aberrant activation of this pathway [22]. Therefore, it is important to determine if LGR6 interacts with the different RSPOS to regulate Wnt/β-catenin signaling and whether the cancer mutations affect LGR6-mediated signaling. In this study, we have characterized the binding and activation of LGR6 by RSPOs. To gain a better understanding of the function of LGR6 in Wnt/β-catenin signaling and oncogenesis, we also evaluated the activity of the different LGR6 mutants identified in colon cancer. Here we show that one of the mutants is incapable of binding to R-spondins and fails to activate Wnt/β-catenin signaling.

## Results

### R-Spondin binds to and co-internalizes with LGR6

**Figure 1:**
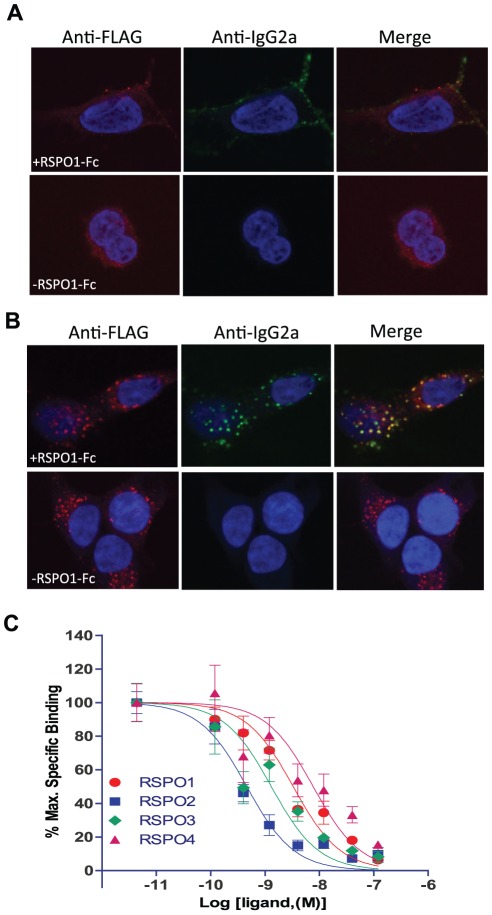
Binding of R-spondins to LGR6 by confocal immunofluorescence analysis and competition binding assay. A&B, HEK293 cells stably expressing Flag-LGR6 were incubated with mRSPO1-Fc or control conditioned media (CM) at 4°C (A), or at 37°C (B). Flag-LGR6 was detected with a Cy3-labeled anti-Flag antibody (red) and mRSPO1-Fc was detected using Alexa488-labeled anti-IgG2a antibody (green). Nuclei were counterstained with To-Pro-3 (blue). The data shown are from one of three independent experiments with similar results. C, Quantitative binding analysis using a whole-cell-based assay. HEK293 cells stably expressing Flag-LGR6 were incubated with mRSPO1-Fc plus serial dilutions of purified recombinant RSPO1–4. Maximum specific binding is defined by the difference between the data with and without mRSPO1-Fc, which is ∼50% of total binding in general. All error bars are SEM (n = 3).

Previously, we reported that R-spondins bind to and co-internalize with LGR4 and LGR5 using immunofluoresence and whole cell binding analysis [9]. We used the same methods to examine if they can also bind to and co-internalize with LGR6. HEK293 cells stably expressing human LGR6 with a Flag-tag at the N-terminus were generated and used in the binding study. A fusion protein consisting of mouse RSPO1 with mouse IgG2a-Fc at the C-terminus (designated mRSPO1-Fc) was shown to be biologically active [23] and used here as a probe. When mRSPO1-Fc was incubated with live cells expressing LGR6 at 4°C to prevent internalization, a strong signal was detected using an anti-IgG2a antibody (Fig. 1A, upper mid panel) while no signal was observed in the absence of mRSPO1-Fc (Fig. 1A, lower mid panel). Co-staining with an anti-Flag antibody showed strong receptor expression in both cases (Fig. 1A, upper and lower left panels). No binding of mRSPO1-Fc to vector control cells could be detected [9]. When the binding was performed at 37°C with live cells, both LGR6 (red) and mRSPO1-Fc (green) were observed in intracellular bodies (Fig. 1B, upper left and mid panels), indicating that both mRSPO1-Fc and LGR6 were internalized. Superimposing of the two images revealed near 100% co-localization of mRSPO1-Fc with LGR6 (Fig. 1B, upper right panel). Again, no anti-IgG2a signal was detected in the absence of mRSPO1-Fc (Fig. 1B, lower mid panel), or in vector control cells [9]. It should be mentioned that intracellular staining of LGR6 was also observed in the absence of mRSPO1-Fc (Fig. 1B, lower left panel), suggesting that a significant portion of LGR6 was internalized by either constitutive activity or endogenous expression of RSPOs in HEK293 cells [9], [12], [24]. We then used competition binding analysis to determine the affinities of the four RSPOs in binding to LGR6. As shown in Figure 1C, all RSPOs are able to displace the binding of mRSPO1-Fc completely. The IC50s displayed by RSPO1, 2, 3, and 4 under these conditions are 3.3, 0.5, 1.7, and 7 nM, respectively. Taken together, these results show that RSPO1–4 bind to LGR6 strongly and specifically, with RSPO2 showing the highest affinity. Furthermore, the affinity profile of LGR6 for RSPO1–4 is highly similar to that of LGR5 [9].

### Stimulation of LGR6 with RSPO1–3 enhances Wnt/β-catenin signaling and this activity is inhibited by Dkk1

**Figure 2:**
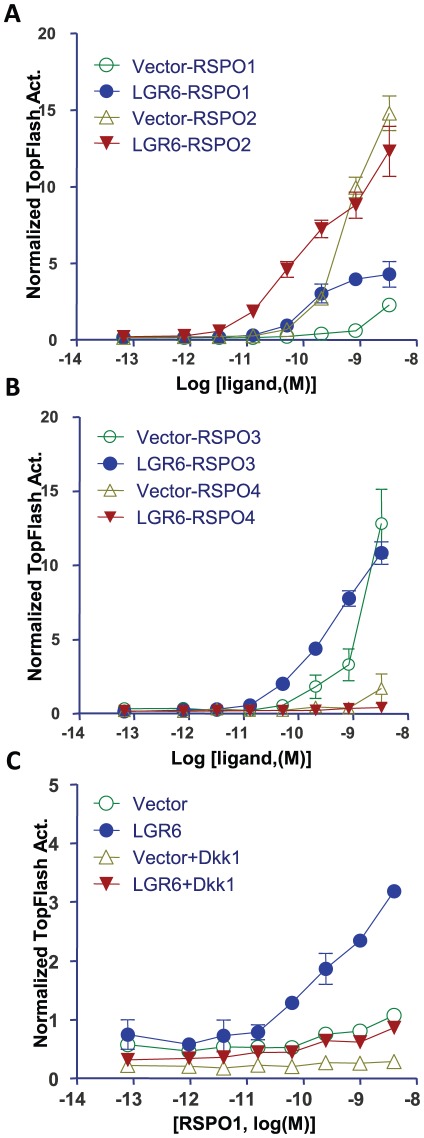
LGR6 enhances Wnt/β-catenin signaling in response to RSPO1–3 and this activity is inhibited by Dkk1. HEK293T cells were transiently transfected with LGR6 or vector control, plus Dkk1 expression plasmids as indicated, along with the β-catenin reporter plasmid super 8× TOPFlash (firefly luciferase) and pRL-SV40 (renilla luciferase) and then stimulated with serial dilutions of purified recombinant RSPO1–2 (A), RSPO3–4 (B) or RSPO1 (C) in the presence of Wnt3a CM. Firefly luciferase activity of each well was normalized to that of renilla luciferase activity of the same well. All error bars are SEM (n = 4).

Since RSPOSs are well known to enhance β-catenin signaling in a Wnt-dependent manner [12], [13], [16], [25], [26], we next investigated whether LGR6 can affect the activity of RSPO in this pathway. Activation of Wnt/β-catenin signaling is routinely measured by monitoring the activity of the Super TOPflash construct which contains a reporter enzyme under the control of 8 TCF/LEF β-catenin-responsive elements [27], [28]. Using the Super TOPflash assay, we examined the effect of R-spondin treatment on Wnt signaling in HEK293T cells overexpressing LGR6 in the presence of low concentrations of exogenous Wnt3a which is required for R-spondin to function [25]. Overexpression of LGR6 increased the potency of RSPO1 by ∼30-fold when compared to vector-transfected cells (Fig. 2A). For RSPO2, strong, endogenous response was observed in vector cells, which was further enhanced upon transfection of LGR6 as shown by the increased potency of the ligand (Fig. 2A). A similar increase in potency was also found for RSPO3 (Fig. 2B). On the other hand, overexpression of LGR6 had no effect on the activity of RSPO4 even though RSPO4 showed high affinity binding to LGR6 (Fig. 1C and Fig. 2B). Previously, it was demonstrated the endogenous response to RSPOs in HEK293T cells are predominantly mediated by LGR4 [8]–[10]. Overexpression of LGR4 or LGR5 in these cells still led to dramatic increase in the potencies of RSPO1–4 (∼1000-fold in the case of RSPO2) [9]. LGR6 binds RSPO1–4 with affinities similar to those of LGR5, but showed much less dramatic effect on the potencies of the four ligands in the Wnt/β-catenin signaling assay. These results suggest that LGR6 is intrinsically weaker in potentiating Wnt/β-catenin signaling following RSPO stimulation. Alternatively, RSPO binding to LGR6 may lead to activation of other signaling pathways yet to be identified.

Dickkopf 1 (Dkk1) antagonizes canonical Wnt signaling by competitively binding to the Wnt ligand binding site of LRP6. This prevents the activity of RSPOs, since co-stimulation with a Wnt ligand is required for RSPOs [24], [29]–[34]. We then tested the effect of Dkk1 on the LGR6-mediated increase in the activity of RSPO1, since the RSPO1 ligand exhibited the weakest endogenous response in HEK293T cells (Fig. 2A). Cotransfection of Dkk1 completely blocked RSPO1 signaling in vector cells as well as in LGR6-transfected cells (Fig. 2C), similar to what was observed with LGR4 and LGR5 [9]. These data suggest that LGR6 functions in a similar mechanism to that of LGR4/5 in mediating RSPO-induced potentiation of Wnt/β-catenin signaling.

### LGR6 is not coupled to heterotrimeric G proteins or to β-arrestin following RSPO stimulation

**Figure 3:**
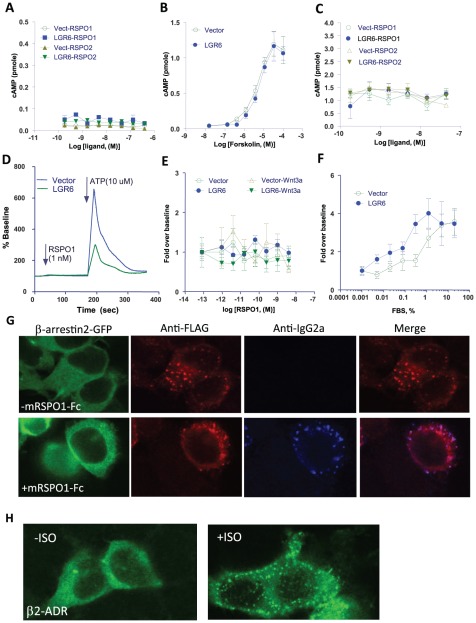
LGR6 is not coupled to heterotrimeric G proteins or to β-arrestin following R-spondin stimulation. A, No cAMP response was detected in vector and LGR6 cells treated with RSPO1–2. B, Forksolin showed a strong stimulation of cAMP production in both cells. C, RSPO1–2 treatments had no effect on forskolin-stimulated cAMP production in vector and LGR6 cells. D, No Ca^2+^ mobilization was induced in vector and LGR6 cells in response to RSPO1, whereas ATP gave a robust response in both cells. E, Stimulation of LGR6 and vector cells by RSPO1 had no effect in the Gα_(12/13)_ pathway using the serum response factor reporter enzyme assay. F, FBS gave a dose-dependent response in the serum response factor reporter enzyme assay. G, No translocation of β-arrestin was observed in LGR6 cells treated with mRSPO1-Fc, whereas colocalization of mRSPO1-Fc with LGR6 was confirmed. H, Robust translocation of β-arrestin was observed in HEK293 cells transfected with β2-adrenergic receptors and stimulated with its agonist isoproterenol.

Since LGR4–6 contain a 7TM domain typical of rhodopsin-like GPCRs, they are thus predicted to be coupled to heterotrimeric G proteins for signal transduction [1]. Heterotrimeric G proteins are classified into four groups, Gαs(stimulation of cAMP production), Gα(i/o) (inhibition of cAMP production), Gαq, (Ca^2+^ mobilization), and G_α(12/13)_ (Rho activation) [35]. In addition, nearly 90% of GPCRs induce β-arrestin translocation for receptor desensitization and alternative signaling [36]. Previously, we demonstrated that stimulation of LGR4 or LGR5 with RSPOs did not induce changes in any of the pathways typically associated with GPCR activation, i.e., cAMP alteration, Ca ^2+^ mobilization, or β-arrestin translocation [9]. We therefore investigated if this is also true for LGR6. First, we examined cAMP response in cells transiently transfected with vector control or LGR6 after treatment with various concentrations of RSPO1–2. No hint of increased cAMP production in either control or LGR6 cells was observed (Fig. 3A). As a positive control, these cells showed a normal, robust increase in cAMP levels in response to forskolin stimulation (Fig. 3B). We then tested if LGR6 inhibits forskolin-stimulated cAMP production, but found no such activity at any of the tested concentrations of RSPO1 or RSPO2 (Fig. 3C). Analysis of Ca^2+^ mobilization also failed to detect any LGR6-mediated activity (Fig. 3D). We also examined if activation of LGR6 is coupled to the Gα_(12/13)_ pathway using a serum response factor-based reporter enzyme assay, which is one of the standard methods for monitoring activation of this pathway [37]. Cells transfected with either vector control or LGR6 showed no response to RSPO1 in this assay, with or without Wnt3a co-treatment (Fig. 3E). Treatment with fetal bovine serum (FBS), as expected, resulted in a dose-dependent increase in reporter enzyme activity (Fig. 3F). LGR6-transfected cells showed a moderate increase in the potency of serum, the significance of which needs further investigation. Lastly, we investigated if β-arrestin is involved in LGR6 activation. No translocation of β-arrestin was detected in HEK293T cells transfected with vector or LGR6 following treatment with RSPO1 (Fig. 3G). LGR6 expression and ligand-receptor co-localization were clearly confirmed (Fig. 3G). As positive control, cells expressing β2-adrenerigc receptor showed robust translocation of β-arrestin following stimulation with its agonist (Fig. 3H). Furthermore, co-stimulation with recombinant Wnt3a in any of the above GPCR assays made no difference. Taken together, these data indicate that LGR6, like LGR4 and LGR5, is not coupled to any of the heterotrimeric G protein classes or to β-arrestin, at least when stimulated by RSPOs.

### LGR6 mutation identified in colon cancer has loss of function

**Figure 4:**
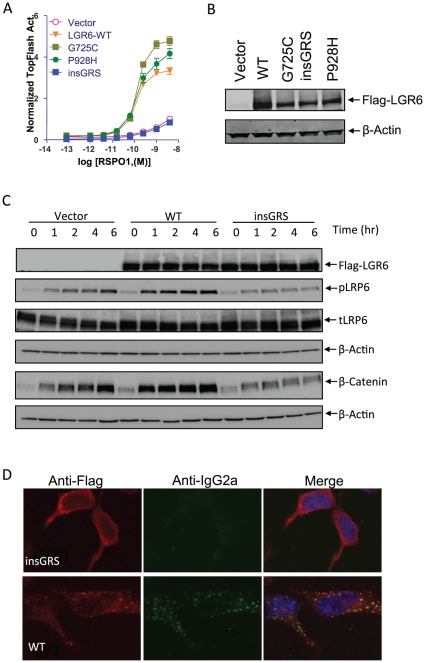
Functional and binding analyses of LGR6 mutants. A, TOPFlash assay of LGR6 mutants on RSPO1-induced potentiation of Wnt/β-catenin signaling. HEK293T cells were transiently transfected with expression plasmids as indicated with a combination of super 8× TOPFlash and pRL-SV40 reporter gene constructs, and then stimulated with serial dilutions of purified recombinant RSPO1 in the presence of Wnt3a CM. All error bars are SEM (n = 4). B, Expression levels of LGR6 mutants and WT in HEK 293T cells in transient transfection paradigms. Total cell lysates were treated with Laemmli buffer for 1 hr at 37°C, fractionated by SDS-PAGE using 4–20% gels, electrophoretically transferred to nitrocellulose membrane, and then probed with anti-Flag antibody. The signal was detected by ECL Western blotting detection reagents (Amersham Biosciences). β-actin was also probed as loading control. C, Time course of changes in Wnt3a-RSPO1-induced β-catenin accumulation and LRP6 phosphorylation in vector, LGR6-WT and insGRS-overexpressing cells. HEK293 cells stably expressing vector, LGR6-WT or insGRS were stimulated with 1 ng/ml RSPO1 plus Wnt3a CM for 0–6 hrs. Total cell lysates were probed with antibodies against Flag-LGR6, phosphor-Ser1490, total LRP6, and β-actin. For the analysis of nonmembrane-bound β-catenin, the cell lysates were cleared with ConA-sepharose beads and then probed with an antibody against β-catenin as described before [9]. D, mRSPO1-Fc binding to LGR6-insGRS and WT. HEK293 cells stably expressing Flag-LGR6-WT or insGRS were incubated with mRSPO1-Fc at 37°C for 1 hr. The cells were fixed, permeabilized, and then co-stained with anti-Flag (red) and anti-IgG2a (green) antibodies to detect LGR6 and mRSPO1-Fc, respectively. Nuclei (blue) were counterstained with To-Pro-3.

Total exon or complete genomic sequencing has increasingly been used to identify all mutations of cancer genomes in a nonbiased fashion [17], [38]. In the very first sequencing of total exons from breast and colon cancer samples, LGR6 was found to be one of the commonly mutated genes in colon cancer with three mutations found in a total of 37 cases [17]. Two of the three mutations are missense and the 3^rd^ one was an inframe insertion of three amino acid residues (Table 1) [17]. Since then, one additional mutation was found in pancreatic cancer and two in ovarian cancer (Table 1) [21], [39]. However, the consequences of these mutations, with one exception, have never been determined since no functional assay was available until now. The frameshift mutation (230fs*6) found in ovarian cancer truncates 737 out of 967 amino acids and is thus expected to be a loss-of-function mutation. With the finding that LGR6 functions as a receptor of RSPOs to potentiate Wnt/β-catenin signaling, we set out to determine the activity of the three mutants identified in colon cancer by comparing them with LGR6 WT. The three mutants and LGR6 WT, along with vector control, were transfected into HEK293T cells and their responses to RSPO1 were compared side-by-side. The two point mutation mutants, G725C and P928H, showed increased responses that are not significantly different from WT (Fig. 4A), implying that they do not affect RSPO1-simulated LGR6 activity. The response curve of the insGRS mutant, however, was indistinguishable from that of vector control (Fig. 4A), suggesting that it has lost the function of responding to RSPO1 in potentiating Wnt/β-catenin signaling. Immunoblot analysis showed all receptors were expressed at a similar level (Fig. 4B), indicating no defects in expression.

**Table 1:** Somatic cancer mutations of LGR6 listed in the COSMIC database as of Jan-2012.

Cancer	Mutation	Affected Domain	Zygosity	Mutated/Total Cases (%)
Colon (adenocarcinoma)	P928H	C-terminal tail	Homozygous	3/37 (8%)
	G725C	Extracellular loop 2	Heterozygous	
	S299insGRS	ECD	Heterozygous	
Pancreas	V746I	TM5	Heterozygous	1/23 (4%)
Ovary (serous carcinoma)	F230fs*6	ECD	Heterozygous	2/3 (67%)
	D536G	ECD	Heterozygous	

Next, we investigated potential defects of the insGRS mutant by comparing its activities with those of WT with respect to LRP6 phosphorylation, accumulation of non-membranous β-catenin, and ligand binding. HEK293T cells were transiently transfected with vector control, LGR6WT, or insGRS plasmids and treated with RSPO1 for different periods of time (0 to 6 hrs) before being harvested for analysis. In vector control cells, RSPO1 stimulation increased LRP6 phorphorylation and accumulation of β-catenin as expected (Fig. 4C). In LGR6 transfected cells, phosphorylation was further increased across all time points (Fig. 4C), consistent with the increased activity in the Super TOPflash reporter enzyme assay. In LGR6-insGRS-transfected cells, both LRP6 phosphorylation and β-catenin accumulation were reduced at later time points when compared to vector control cells (Fig. 4C), indicating the mutant is defective in enhancing Wnt3a-stimulated signaling. Again, the expression level of the mutant was similar to that of the WT receptor based on western blot analysis (Fig. 4C). We then performed ligand binding analysis to determine if the LGR6-insGRS receptor is still capable of binding to RSPO1, especially since the mutation is located in the presumed ligand-binding ECD. When the binding was carried out at 37°C, LGR6-insGRS-expressing cells showed receptor staining at both the cell surface and in the cytoplasmic space (Fig. 4D, upper left panels). However, no staining of mRSPO-Fc was observed, indicating that the mutant is incapable of binding to the ligand. LGR6-WT-expressing cells showed strong staining of the ligand which is co-localized with the receptor (Fig. 4D, lower panels). Furthermore, examination of the intracellular staining pattern of the insGRS mutant found no vesicular structures typically observed with LGR4–6 (Fig. 4A, left panels) [9]. This suggests that the mutant does not undergo normal receptor internalization, potentially due to its failure to bind ligand. The strong, non-vesicular intracellular staining also suggests that the mutant is potentially defective in trafficking to the membrane after synthesis in the ER. Collectively, these data indicate that RSPO1 stimulation of LGR6-WT enhanced Wnt-induced LRP6 phosphorylation and β-catenin accumulation, leading to increased Wnt signaling output. Of the three mutants found in colon cancer through random sequencing, only the insGRS mutant has loss of function. This loss of function is due to its inability to bind ligand, which may be attributed to a conformational change in the receptor as a consequence of the insertion of three amino acid residues in the ECD's leucine-rich repeat #10. Together with the finding that LGR6 is hypermethylated in up to 50% of colon cancer samples and an LGR6 truncation mutant has been identified in ovarian cancer, these data imply that LGR6 functions as tumor suppressor for colon and ovarian cancer.

### Overexpression of LGR6 increases cell migration

**Figure 5:**
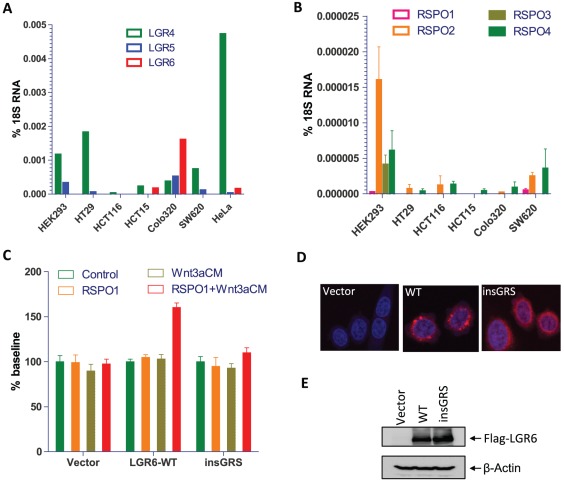
Expression profiling of LGR4–6 and RSPO1–4 and migration assay of HeLa cells overexpressing LGR6. A, Expression levels of LGR4–6 in HEK293 cells and a panel of cancer cell lines determined by quantitative RT-PCR analysis. B, Expression levels of RPSO1–4 in HEK293 and a panel of colon cancer cell lines determined by qPCR analysis. C, Cell migration analysis of HeLa cells stably expressing vector, LGR6-WT or insGRS after treatments with vehicle control, RSPO1,Wnt3a-CM, or RSPO1+Wnt3a. Data are presented at mean ± S.E.M. of three replicates after normalization to vehicle control. The experiment was repeated once and both showed similar results. D & E, Expression analysis of LGR6-WT and insGRS in HeLa cells by confocal immunofluorescence (D) and immunblotting (E) using an anti-Flag antibody.

To understand potential roles and mechanisms of LGR6 in oncogenesis, we profiled a panel of colon cancer and uterine cancer cell lines for expression levels of LGR4–6 and RSPO1–4 (Fig. 5A–B). Of the three receptors, LGR4 is the most commonly and abundantly expressed receptor in the cancer cell lines, with the exception of the HCT116 cells (Fig. 5A). LGR6 is expressed at negligible levels except in Colo320 cells. Interestingly, none of the colon cancer cell lines expressed significant levels of RSPOs while previous published results showed HeLa cells express high levels of RSPO3 [12] (Fig. 5B). To examine the effect of LGR6 on growth and migration of cancer cells, we attempted to overexpress LGR6 WT and insGRS mutant in SW620 cells which have relative low expression of LGR4–6 and are routinely used as a model of colon cancer studies. However, repeated efforts failed to establish cell lines stably expressing WT or mutant LGR6. We then used HeLa cells as a host cell line and obtained bulk cell lines stably expressing FLAG-tagged LGR6 WT or insGRS after sorting with anti-Flag antibody (Fig. 5 D–E). No difference among the three cell lines expressing vector, LGR6-WT or LGR6-insGRS was observed in growth rate as measured by the xCelligence assay (data not shown). We then compared migration of the three cell lines treated with vehicle control, RSPO1, Wnt3a, or Wnt3a+RSPO1. No significant difference in baseline migration was observed among the three cell lines (Fig. 5C). Interestingly, cells expressing LGR6-WT displayed increased migration when treated with RSPO1+Wnt3a while cells expressing the insGRS mutant cells or the vector control showed no response, confirming the loss of function result for the insGRS mutant. Confocal immunofluoresence analysis showed that the insGRS mutant, like in HEK293 cells, is not located in intracellular bodies (Fig. 5D). In contrast, WT LGR6 consistently displayed a pattern of vesicular staining (Fig. 5D). These results indicate that overexpression of LGR6 in HeLa cells can increase cell migration when co-stimulated by Wnt3a and RSPO1.

## Discussion

In the present study we determined the affinity and potency of LGR6 for RSPO1–4 in potentiating Wnt/β-catenin signaling, and characterized the activity of three mutations identified in colon cancer samples. Compared to LGR4 and LGR5, LGR6 appears to have a more limited tissue distribution with lower expression in general (http://www.ncbi.nlm.nih.gov/UniGene/ESTProfileViewer.cgi?uglist=Hs.497402). Knockout of LGR6 in the mouse has no obvious phenotype while homozygous deletion of either LGR4 or LGR5 leads to total embryonic or neonatal lethality [3], [40], [41]. As loss of RSPOs leads to embryonic lethality or major developmental defects [42], these observations suggest that LGR6 does not have a major role in mediating the effect in RSPOs in vivo. Interestingly, our in vitro analysis substantiates that LGR6 is much less effective in potentiating RSPO-mediated Wnt/β-catenin signaling as shown by the much less dramatic increase in the potencies of RSPO1–4 when compared to LGR4/5, even though recombinant expression of LGR6 gave comparable receptor levels and binding affinities for RSPO1–4. Assuming the 7TM domain of LGR4–6 is responsible for receptor signaling, we compared the 7TM sequence of LGR6 with those of LGR4 and LGR5, and found that LGR6 is unique in one potentially important motif. The “DRY” motif located at the end of TM3, of which the Arg residue (often referred to as residue 3∶50) is almost absolutely conserved in the rhodopsin family of GPCRs. In LGR4 and LGR5, the sequence is “ERG” and “ERS”, respectively. In LGR6, however, the sequence is “QCS”, representing one of the rare cases of receptors without an Arg residue at the 3∶50 position in the rhodopsin family. This deviation may be one of the factors for the decreased efficacy of LGR6 in potentiating Wnt/β-catenin signaling.

Similar to LGR4 and LGR5, LGR6 is not coupled to heterotrimeric G proteins or to β-arrestin following stimulation with RSPO alone or RSPO plus Wnt3a. This conclusion is based on our analysis of 2^nd^ messengers, Gα_(12/13)_-induced transcriptional activation, and β-arrestin translocation. The observation that HEK293T cells showed no hint of endogenous response to RSPO1 or SPO2 in the Gα_(12/13)_ assay also indicates that LGR4 is not coupled to this pathway since these cells have strong endogenous expression of LGR4 and respond to RSPO1 stimulation in the Wnt/β-catenin signaling assay [8], [9]. Though the 7TM region of LGR4–6 has significant overall homology to those of rhodopsin-like GPCRs and contains all the important motifs, it does have unique features that may hold the answer to their lack of coupling to G proteins. The intracellular loop between TM5 and TM6 in GPCRs directly interacts with the α subunit of the heterotrimeric complex [43], and generally has an overall basicity (pI = ∼9). For LGR4–6, however, the loops have a calculated pI of ∼5.5, which can potentially hinder interactions with G proteins. The CWXP motif in TM6, commonly conserved in the rhodopsin family of receptors, has the sequence XXCP for LGR4–6. This motif plays a critical role in the activation of heterotrimeric G proteins [35], [44], [45]. All the other members of the LGR family (LGR1–3 and LGR7–8), which are universally coupled to Gαs and are the most closely related homologs of LGR4–6, have the sequence CMAP (LGR1–3) or CWIP (LGR7–8) for this motif [46]. The Drosophila receptor dLGR2, a Gαs-coupled receptor for the neuropeptide bursicon [47], [48], is most homologous to LGR4–6 and was believed to be the invertebrate ortholog of mammalian LGR4–6 [49]. Notably, dLGR2 has the sequence CWSP for this motif. Interestingly, orthologs of RSPOs have not been identified in invertebrates and bursicon-like mammalian peptides such as gremlins don't not activate LGR4–6 [9]. Therefore, we speculate that LGR4–6 have evolved away from their typical ancestor GPCRs to function as receptors for the newly evolved RSPO proteins to potentiate Wnt/β-catenin signaling in vertebrates. In fact, several regulators of the Wnt signaling pathways are only found in vertebrates, such as DKK1 and WTX, both of which inhibit canonical Wnt signaling [50], [51]. The finding of a vertebrate-specific LGR-RSPO receptor ligand system being uncoupled to heterotrimeric G proteins is rather surprising, but perhaps not unexpected since they are the only receptors of this family that are specifically associated with stem cell function among the hundreds of 7TM rhodopsin-like receptors. Delineation of the signaling mechanisms of LGR4–6 will provide important insights to the function of these receptors and the control of proliferation and differentiation of adult stem cells.

LGR4–6 are all known to be associated with various types of cancer, but their roles and mechanisms in oncogenesis remain largely unknown. LGR4 expression was shown to be increased in approximately half of colon cancer cases with high levels being associated with more severe metastasis [52]. For LGR5, earlier studies suggest that it has increased expression in colon cancer and basal cell carcinoma with expression potentially enriched in cancer stem cells [53]–[55]. More recent publications, on the other hand, reported that LGR5 may actually function as a tumor suppressor and its expression level is inversely correlated with prognosis [56], [57]. Somatic cancer mutations of LGR5 have also been identified, all of which are distinct missense mutations with the exception of one truncation [21], supporting its role as a tumor suppressor. For LGR6, the initial evidence came from the finding of three mutations in random, total exon sequencing of 37 colon cancer samples [17]. A transcriptome-wide approach showed that LGR6 is hypermethylated in the promoter region in ∼20% of colon cancer [18], and a follow-up study found hypermethylation in up to 50% of colon cancer samples depending patient ethnicity [20]. Our analysis of the three mutations found in colon cancer clearly indicates that one of them (insGRS) is a loss-of-function mutation. Based on these data, it is suggested that LGR6 plays the role of a tumor suppressor in colon and ovarian cancer. Interestingly, RSPO1, a high affinity ligand of LGR6, also appears to function as a tumor suppressor. Loss of RSPO1 in females recessively leads to increased risk of squamous cell carcinoma [58]. The notion that LGR6 is expressed specifically in a population of stem cells that can give rise to all cell lineages of the skin and that cancer often originates from stem cells suggests that LGR6 may be the underlying receptor for the tumor suppressive role of RSPO1. On the other hand, the finding that LGR6 has positive effects on Wnt/β-catenin signaling and cell migration appears to be inconsistent with a tumor suppressor function. It is generally believed that hyperactivation of Wnt/β-catenin signaling leads to increased cell proliferation and cell migration is important for metastasis, both of which are expected to have oncogenic function. However, emerging evidence suggests that Wnt/β-catenin signaling has to be kept at an appropriate (“just right”) level to balance proliferation, differentiation and apoptosis [59], [60]. Thus, suppression of LGR6 function may be important for cancer cells to maintain the right level of Wnt/β-catenin signaling. As for cell migration, the effect of LGR6 may be specific to HeLa cells. LGR5, for example, showed oncogenic properties in colon cancer cells and basal cell carcinoma cells [53], [54], but displayed tumor suppressor function in colon cancer cell lines with β-catenin mutations [56]. Interestingly, the tumor sample containing the LGR6 insGRS mutation also has mutation in APC but not in β-catenin [21], suggesting that the action of LGR6 does not depend on the genetic status of β-catenin. Nevertheless, the finding of loss-of-function mutations in cancer cells and promoter hypermethylation strongly argues that LGR6 functions as a tumor suppress in colon and ovarian cancer.

In summary, we characterized the binding and functional activities of RSPO1–4 on LGR6 and found that LGR6 has the highest affinity for RSPO2. Stimulation of LGR6 with RSPO1 leads to increased LRP6 phosphorylation and β-catenin-controlled transcriptional activity. We also determined the activity of three somatic mutations found in colon cancer and demonstrated that the insGRS mutant is a loss-of-function mutation due to failure of ligand binding. Overexpression of LGR6 in HeLa cells leads to increased cell migration in response to treatment with Wnt3a and RSPO1. These results suggest that LGR6 is potentially a tumor suppressor and provide a basis for future research into the roles and mechanisms of LGR6 in oncogenesis.

## Materials and Methods

### DNA Constructs and Recombinant Proteins

A plasmid containing the full-length open reading frame (ORF) of human LGR6 was purchased from Open Biosystems. The sequence encoding a predicted mature form of human LGR6 (AA25–967, Genbank accession number NP_001017403) was fused with a sequence encoding a Flag tag at the N terminus, and then cloned downstream of a sequence encoding the CD8 signal peptide (MALPVTALLLPLALLLHAA) in the vector pIRESpuro3 (Clontech) using standard, PCR-based molecular cloning procedures. LGR6 mutants were created from this wildtype (WT) construct using the QuikChange site-directed mutagenesis kit (Strategene, La Jolla, CA). The sequences of the primers used for site-directed mutagenesis are listed in Table 2. The entire coding regions of the mutant plasmids were bi-directionally sequenced to verify that there were no errors introduced during PCR. All other reagents and plasmids were as described previously [9].

**Table 2:** Primers used for the creation of mutant hLGR6 by site-directed mutagenesis.

Mutation	Primers
InsGRS (F)^a^	5′-GTGGGAAGATCGG**GAAGATCGG**CATTCCAGTACCTGC-3′
InsGRS (R)	5′-GCAGGTACTGGAATG**CCGATCTTC**CCGATCTTCCCAC-3′
G725C (F)	5′-CTACGCGCCACCTGAG**T** GTCAGCCAGCAGCCCTG-3′
G725C (R)	5′-CAGGGCTGCTGGCTGAC**A**CTCAGGTGGCGCGTAG-3′
P928H (F)	5′-CCACTTTGGGAACC**A**CCAACCCTCCATGG-3′
P928H (R)	5′-CCATGGAGGGTTGG**T**GGTTCCCAAAGTGG-3′

a F and R in the parentheses denote forward and reverse primers, respectively. The codons encoding the amino acid substitutions or insertion are underlined and indicated in bold typeface.

### Binding and functional analysis of LGR6 WT and mutants

Cell culture and transfection of HEK293 and HEK293T cells (purchased from ATCC) were carried out as described previously [9]. Binding of mRSPO1-Fc to cells expressing LGR6 at 4°C and 37°C were performed as before [9], except that Cy3-labeled anti-Flag antibody was used. Measurements of cAMP levels, Ca^2+^ mobilization, and β-arrestin translocation were carried out side-by-side with LGR4 and LGR5 previously [9]. For the analysis of the Gα_(12/13)_ coupling pathway, HEK293T cells were transiently transfected with 1 µg of SRF-RE-*luc2P* reporter plasmid (Promega) and 100 ng of pRL-SV40 plasmid together with 1 µg of LGR6-WT or vector control. Twenty four hours after transfection, the cells were detached, seeded and serum starved overnight in a 384-well plate. Luciferase activity was measured after six hour induction with serial dilutions of either RSPO1 or FBS using the Dual-Glo™ assay system (Promega) according to the manufacture's protocol.

### Immunoblot Analysis

Immunoblotting of phosphorylated LRP6, total LRP6, and cytosolic (nonmembrane bound) β-catenin were performed as described previously [9]. Immunoblot analysis of Flag-LGR6 WT and mutants were carried out using an anti-Flag (1∶1000; Sigma) using standard conditions.

### Expression analysis of LGR4–6 in cancer cell lines and cell migration assays

All cancer cell lines were grown in DMEM+10% FBS in a 37°C incubator with 5% CO_2_ and 95% humidity. RNA isolation and quantitative PCR analysis were carried out using primers and conditions described previously [9]. The sequences of forward and reverse primer used for LGR6 are *5′-*CTCTTCCCTTTCCTCTC-3′ and 5′- CTGAGTTTTGGTTGTATTTG-3′, respectively. For the generation of HeLa cells stably expressing LGR6WT, mutant, or vector control, the corresponding plasmids were transfected into HeLa cells with Fugene HD and selected with puromycin (1 µg/ml). Drug-resistant cells from LGR6WT or mutant transfected cells were sorted for receptor expression using a Cy3-labeled anti-FLAG antibody. For cell migration assay, HeLa cells stably expressing control vector, LGR6-WT or mutant were studied in a permeable filter of transwell system (BD BioCoat™ Control 8.0 µm PET Membrane 24-well Cell culture inserts, BD Biosciences). After trypsinization, cells was seeded at 1×10^4^ cells/well into the upper chamber which contains culture medium with 1% FBS. Cell migration to the other side of membrane was induced by 10% FBS-containing medium in the lower chamber for 48 h at 37°C in a tissue culture incubator. Migrated cells in the lower chamber were fixed in 4% PFA solution for 10 min, stained in 0.03% crystal violet solution for 10 min, and then rinsed in water. The stained cells were subjected to microscopic examination on a light microscope. Migrated cells were counted in ten randomly selected fields per insert, and the values were averaged. All experiments were performed at least twice with three replicates under each migration condition.
